# Barriers, facilitators and needs to deprescribe benzodiazepines and other sedatives in older adults: a mixed methods study of primary care provider perspectives

**DOI:** 10.1186/s12877-024-05027-9

**Published:** 2024-05-04

**Authors:** Orlando Hürlimann, Daphne Alers, Noël Hauri, Pascal Leist, Claudio Schneider, Lucy Bolt, Nicolas Rodondi, Carole E. Aubert

**Affiliations:** 1 Department of General Internal Medicine, Inselspital, Bern University Hospital, University of Bern, Anna-Von-Krauchthal Weg 7, Bern, 3010 Switzerland; 2 Institute of Primary Health Care (BIHAM), University of Bern, Bern, Switzerland

**Keywords:** Benzodiazepine, Deprescribing, Mixed methods, Older adults, Primary care, Qualitative, Sedative, Sleep disorder

## Abstract

**Background:**

Benzodiazepines and other sedative hypnotic drugs (BSHs) are frequently prescribed for sleep problems, but cause substantial adverse effects, particularly in older adults. Improving knowledge on barriers, facilitators and needs of primary care providers (PCPs) to BSH deprescribing could help reduce BSH use and thus negative effects.

**Methods:**

We conducted a mixed methods study (February-May 2023) including a survey, semi-structured interviews and focus groups with PCPs in Switzerland. We assessed barriers, facilitators and needs of PCPs to BSH deprescribing. Quantitative data were analyzed descriptively, qualitative data deductively and inductively using the Theoretical Domain Framework (TDF). Quantitative and qualitative data were integrated using meta-interferences.

**Results:**

The survey was completed by 126 PCPs (53% female) and 16 PCPs participated to a focus group or individual interview. The main barriers to BSH deprescribing included patient and PCP lack of knowledge on BSH effects and side effects, lack of PCP education on treatment of sleep problems and BSH deprescribing, patient lack of motivation, PCP lack of time, limited access to cognitive behavioral therapy for insomnia and absence of public dialogue on BSHs. Facilitators included informing on side effects to motivate patients to discontinue BSHs and start of deprescribing during a hospitalization. Main PCP needs were practical recommendations for pharmacological and non-pharmacological treatment of sleep problems and deprescribing schemes. Patient brochures were wished by 69% of PCPs. PCPs suggested the brochures to contain explanations about risks and benefits of BSHs, sleep hygiene and sleep physiology, alternative treatments, discontinuation process and tapering schemes.

**Conclusion:**

The barriers and facilitators as well as PCP needs and opinions on patient material we identified can be used to develop PCP training and material on BSH deprescribing, which could help reduce the inappropriate use of BSHs for sleep problems.

**Supplementary Information:**

The online version contains supplementary material available at 10.1186/s12877-024-05027-9.

## Background

Benzodiazepines and other sedative hypnotic drugs (BSHs) are frequently prescribed for sleep problems, although guidelines recommend cognitive behavioral therapy for insomnia (CBT-I) as first-line therapy and to avoid BSHs in older people [1, 2]. BSH use has been reported to be as high as 15–30% in older adults with 87% taking BSHs for sleep problems [3–5]. Nevertheless, their effects on sleep are only modest and short-lasting [6, 7]. Furthermore, BSHs cause relevant side effects, including falls, fractures and cognitive impairment as well as dependence [8, 9]. This leads to high healthcare and social costs [10]. Moreover, this also highlights a need for deprescribing.

Deprescribing is defined as “the process of withdrawal of an inappropriate medication, supervised by a health care professional with the goal of managing polypharmacy and improving outcomes” [11]. Barriers and facilitators to BSH deprescribing from the perspective of patients, physicians and nurses have been studied previously and the application of the Theoretical Domains Framework (TDF) allowed identification of different behavioral determinants to BSH deprescribing in the ambulatory setting [12–14]. Moreover, different interventions to deprescribe BSHs have been investigated and shown variable success [15]. This variability might be due to a lack of consideration of barriers and facilitators, among other at a local level. While some barriers and facilitators are universal, others might indeed be more specific to the context.

To our knowledge, barriers and facilitators to deprescribe BSHs in older adults have not been studied in Switzerland. Furthermore, primary care providers (PCPs) needs and opinions on what could support them and their patients have not been evaluated.

The aim of our study was thus to identify local barriers and facilitators to deprescribe BSHs and further assess PCPs perspectives on what could support them and their patients in deprescribing BSHs.

## Methods

### Study design and setting

We conducted a mixed methods parallel study including a survey, semi-structured interviews and focus groups (FGs) with PCPs in Switzerland. The survey included both quantitative and qualitative questions, while interviews and FGs collected qualitative data. A mix of interviews and FGs was chosen for practical organizational reason (easier to plan individual interviews than FGs with PCPs). Focus groups are used in qualitative research and allow to explore thoughts and concepts of participants during a group discussion led by a researcher. The FGs and interviews took place in March 2023, and the survey was open from February to May 2023.

The study protocol was waived from approval by the local ethical committee (“Kantonale Ethikkommission Bern”), because it did not fall under the Swiss Human Research Law (request number 2022–01423). Participation was voluntary, and participants provided consent for interview/FG recording. They were informed that their name would appear nowhere.

This article follows the STROBE checklist for reporting [16].

### Study population and sample size

For the survey, PCPs working in an ambulatory practice in any part of Switzerland and caring for an adult population (i.e., not pediatrician) were eligible. For the interviews and FGs, recruitment was limited to the French- and German-speaking parts of the country, which represent 85% of the population of Switzerland. PCPs were contacted by e-mail, as well as through advertisements in medical journals and newsletters usually read by PCPs in Switzerland.

Using Survey System calculator (www.surveysystem.com/sscalc.htm), we calculated that 120 PCPs would provide a margin of error of 9% with a 95% confidence interval. For the qualitative part, we estimated that we would achieve data saturation with 10 interviews/FGs. We planned about half of them with native French speakers, and the other half with native German speakers. Except for language, other elements for variation of sample (e.g., gender, level of experience) were not specially considered.

### Data collection and study procedures

The survey was conducted online using surveymonkey.com (SurveyMonkey Inc., San Mateo, California, USA) and was available in German and in French. It included open- and close-ended questions on demographic variables and professional experience, as well as on barriers, facilitators and needs to deprescribe BSHs in older adults taking BSHs for sleep problems (Additional File 2). At the end of the survey, PCPs had the possibility to disclose their contact information to be rewarded an amount of CHF20 (about $20) for participation. They were informed that their data would be treated confidentially. However, they could also leave their answers anonymous. This process was introduced to reduce the risk of selecting only PCPs who agreed to disclose their contact information.

The interviews and FGs were conducted virtually and based on a guide available in Additional File 3. The guide was developed by the authors based on the research goals. It included an explanation of the general conditions of interviews/FG conduction and an introduction of the topic, followed by six questions concerning perceived barriers, facilitators and needs to deprescribe BSHs in older adults taking them for sleep problems. Interviews and FGs were led by two researchers (DA and CEA). The choice of conducting FGs or interviews was led by PCP availability in term of dates and time. Additional researchers (OH, PL) assisted and took field notes. A duration of 20 min for the interviews and 45 min for the FGs was planned. PCPs were compensated CHF30 (about $30) for participation. Interviews and FGs were recorded and transcribed verbatim.

### Measures

The survey was divided in four parts: 1) nine questions on demographic parameters including age, gender, practice setting, work status (independent vs. employed), professional experience and daily work routine (having ever discontinued BSHs, routinely considering BSH discontinuation, knowing where to refer patients for treatment of sleep problems and for CBT-I); 2) three questions on the need for material for patients; 3) three questions about the needs of a PCP; and 4) three open questions for adding additional information.

### Data analysis

Quantitative analysis: Survey quantitative data and population characteristics (age, gender, practice setting, work status (independent vs. employed), professional experience and daily work routine (having ever discontinued BSHs, routinely considering BSH discontinuation, knowing where to refer patients for treatment of sleep problems and for CBT-I)) were analyzed using descriptive statistics and presented as numbers with percentages.

Qualitative analysis: Qualitative data were analyzed using a mixed deductive and inductive approach based on the TDF version 1, which is frequently used for deprescribing/de-implementation research [13, 14, 17–19], to identify barriers and facilitators to BSH deprescribing. The TDF version 1 includes the following domains: knowledge; skills; social/professional role and identity; beliefs about capabilities; beliefs about consequences; motivation and goals; memory, attention and decision processes; environmental context and resources; social influences; emotion; behavioral regulation; nature of behaviors. Coding was conducted by PL and NH and iteratively discussed with the senior author, who made final adaptations.

Integration of quantitative and qualitative data: Quantitative and qualitative results were integrated using joint displays to draw meta-inferences. Meta-inferences are conclusions that we can draw of the integration of both qualitative and quantitative data. We used them to describe the mixed data results as convergent, divergent, or expanding.

When quotes were reported in the article, they were translated to English by the first author, while the last author checked the translation. Both authors are fluent in German, French and English.

Quantitative data were analyzed with Stata version Stata/MP 16.0 (StataCorp LP, College Station, Texas) and qualitative data using MAXQDA 2022 software (VERBI Software, Berlin, Germany).

We use the following abbreviations to report qualitative statements: PCP = primary care provider; FG = focus group; I = Interview; S = Survey; F = French; G = German.

## Results

### Study population

The survey was completed by 126 PCPs, including 51 (40.5%) French-speaking and 75 (59.5%) German-speaking participants; 16 of them had been approached by e-mail, the rest answered following the advertisements and newsletters. Eleven additional PCPs completed the baseline characteristic form but did not answer any additional survey question and were thus not kept for analysis. Baseline characteristics are presented in Table 1. Of the 126 PCPs, nine participated to an FG (all of them German-speaking) and seven (six French-speaking, one German-speaking) to an individual interview. Fourteen did not provide contact information. The average duration was 26 min for the interviews and 41 min for the FGs.

**Table 1:** Baseline characteristics (*N* = 126)

VARIABLE	N (%)
**Age**	
≤ 30y	2 (1.6)
31-40y	32 (25.4)
41-50y	37 (29.4)
51-60y	35 (27.8)
≥ 61y	20 (15.9)
**Sex**	
Female	67 (53.2)
Male	59 (46.8)
**PCP practice setting**	
Group practice with PCPs	66 (52.4)
Group practice with PCPs and other HCPs	32 (25.4)
Single practice	24 (19.1)
Other^a^	4 (3.2)
**Experience**	
< 5y	4 (3.2)
5-9y	19 (15.1)
10-14y	23 (18.3)
15-19y	20 (15.9)
≥ 20y	60 (47.6)
**Work status**	
Independent	81 (64.3)
Employed	35 (27.8)
No answer	10 (7.9)

*Abbreviations: HCPs* healthcare professionals (specialized physicians and/or non-physician therapists), *PCPs* primary care providers, *y* years

^a^primary care practice and hospital, nursing home network, not working clinically at the moment, “no answer”

### PCP work routine concerning BSH deprescribing

One hundred and sixteen (92.1%) PCPs had ever discontinued BSHs in older adults with sleep problems and 86 (68.3%) routinely considered doing it. Eighty-six (68.3%) PCPs reported that they knew where to refer their patients for the treatment of sleep problems and 58 (46.0%) knew where to refer patients for CBT-I.

### Barriers and facilitators to BSH deprescribing

In this section, we present the results of the qualitative analysis for which there was no quantitative counterpart, based on the TDF. The TDF domains and constructs that were found during the coding process are displayed in Additional File 1: Appendix Table 3. Below are the domains presented with some examples of the qualitative analysis.

#### Knowledge

This section reports knowledge of PCPs and patients influencing BSH deprescribing, which was identified as a barrier. Regarding patient knowledge, unrealistic sleep expectations of patients were experienced as a major issue [PCP16, I7, G]: *“… the main problem, it seems to me, is that patients simply need to be able to sleep when it gets dark and nothing is going on, to sleep the whole night if possible, and only wake up again when the day begins.”* Furthermore, PCPs mentioned that their patients lacked knowledge about BSH risks [PCP7, FG1, G]: *“Well, so what I find difficult is, hmm, that patients are not aware that these are problematic drugs. (…) Because, hmm, apparently this was either not communicated when they were prescribed or, which is also quite possible, it [the information] was put aside afterwards.”* Regarding knowledge of PCPs, some PCPs expressed a lack of knowledge about side effects of BSHs [PCP15, FG3, G]: *“And what is the evidence regarding the, hmm, harmfulness of these, hmm, Z-substances?”.*

#### Skills

Lack of skills was identified as a barrier. PCPs mentioned they had not received enough training on the treatment of sleep problems and BSH deprescribing [PCP13, FG2, G]: *“So it's such a huge problem. I think it would need some kind of course during medical school. And during residency, hmm, definitely too, or simply that it becomes more important. (…) So I think it has far too little importance. Already during the whole training.”*

#### Motivation and goals

Both barriers and facilitators were classified in this construct. A main barrier encountered by PCPs was patient lack of motivation [PCP2, I2, F]: *“And then when we come to it [discussing BSH deprescribing], well, they don't want to talk about it too much. They avoid the subject and then say, ‘Oh no, but we'll do it next time.’”* PCPs didn’t consider BSH deprescribing as a priority [PCP12, FG2, G]: *“Of course, it [BSH deprescribing] is also time-consuming. So, in the primary care practice, apparently there are on average over four problems per consultation and, hmm, then you have to think about how to use the time. And if there are three much more important problems, then, hmm, you just look at those.”* Regarding facilitators, some physicians reported using side effects to motivate their patients to discontinue or not to start BSHs [PCP5, I5, F]: *“So, I often talk to them about cognitive impairment and the risk of fall. I think these are really important problems for older adults.”* Beside these side effects, patient fear of dependence was mentioned as a facilitator to discontinue or not to start BSHs [PCP4, I4, F]: *“The fear of dependence too, I think that's also something that, that can be a lever.”*

#### Environmental context and resources

Environmental context and resources were identified as barriers. Regarding external factors, PCPs experienced lack of time as a barrier to deprescribe BSHs [PCP8, FG1, G]: *“Hmm, what I miss is simply the setting and the peace and quiet to discuss it [BSH deprescribing] with the patient, because that also takes a lot of time, so the quarter of an hour I have in the agenda is often not enough.”* When coming to the prescription of CBT-I, the limited availability and access to it were mentioned as barriers, making prioritization needed [PCP7, FG1, G]: *“But of course, there are too few therapy places, so they are very quickly booked. And, I have to say, I would almost be a bit reluctant to take up such a place for a simple sleep disorder, because there are really many patients with much bigger problems who need it [cognitive behavioral therapy] more urgently.”*

#### Social influences

Lack of public dialogue about BSHs was identified as a barrier, while PCP thought it could facilitate BSH deprescribing [PCP8, FG1, G]: *“And I also think a social dialogue, that these are addictive substances, would be very helpful, because then, hmm, maybe they [the health authorities] would give me more time to deal with it [BSH deprescribing] in peace, to get away from these addictive substances.”*

#### Emotion

Several barriers were related to emotions. PCPs said patient fear of not being able to sleep was a barrier to deprescribe BSHs [PCP3, I3, F]: *“The first argument, very often, is, ‘No, no, but you can't take away my [BSH] (…) Since I have it, I can sleep. I don't want to disturb that balance. And it's so important for me to sleep, as I've gone through periods with so much insomnia.’”* PCPs reported frustration following repeated failed attempts to deprescribe BSHs to be a barrier to try again [PCP17, S, G]: *“My attempts often or almost always fail. (…) So I don't have the courage to try again.”*

#### Behavioral regulation

Several issues related to behavioral regulation were identified as barriers, while other were rather facilitators. PCPs mentioned that costs could impact patient behavior related to BSH deprescribing [PCP18, S, F]: *“Difficulties in getting patients to come back for follow-up consultations. High deductibles, fear of costs.”* On the other hand, it was perceived positively that in Switzerland CBT-I is now covered by universal health insurance [PCP5, I5, F]: *“So, since, since the, hmm, psychotherapy by psychologists started to be covered by health insurance last year, I've really been trying to guide patients by saying, ‘Well, now you can have twice fifteen sessions with a psychologist. It's covered by insurance.’”* PCPs experienced patient social situation and interests as a barrier to deprescribe BSHs and implement sleep hygiene measures [PCP14, FG3, G]: *“Hmm, but then we end up slipping into complex social difficulties because the problem is, especially in winter: ‘What do you do until eleven in the evening and what do you do at six in the morning?’”.*

#### Nature of the behaviors

Starting deprescribing at hospital was identified as a facilitator [PCP1, I1, F]: *“If, if they [the physicians at hospital] can remove [BSHs], and, in parentheses, prove that in hospital they [the patients] sleep without, hmm, they can, in parentheses, more easily keep building on that momentum.”* Nevertheless, PCPs made the experience that tapering was often not continued by patients after hospital discharge, which could be addressed by improving continuity of follow-up [PCP5, I5, F]: *“… sometimes, when they [the patients] come out of geriatrics, they come out of a unit where there was a lot of motivated people who managed to, supposedly, wean them off benzos. But when the patients come out, well, they run to the pharmacy to get them [the benzodiazepines]. So, hmm, would it also be necessary for a psychologist to be directly involved in the discharge process? To say, “Ah, we're going to support you now that you're going home, to prevent a relapse.” Could it be?”.*

### PCP opinions on patient material

In this section, we present the mixed methods results about PCP opinions on what could support them and their patients to discontinue BSHs. Meta-interferences are presented in Tables 2 and 3 and complete quantitative survey results in Additional File 1: Appendix Tables 1 and 2.

**Table 2:** Meta-interference: PCPs opinions on patient material (*N* = 126)

	**Variable**	**N (%)**	**Reflective quote**	**Meta-interference**
FORMAT	Flyers	62 (49.2)	[PCP4, I4, F]: *“But I'd avoid making leaflets, well, little brochures that are too, hmm, too thick. Sometimes making a, a flyer that fits on one page, on both sides, that could be pretty good.” *	Divergent
			[PCP1, I1, F]: *“I don't know. In the waiting room, well, I have lots of leaflets on lots of things.”*	Convergent
	Brochures	87 (69.1)	[PCP2, I2, F]: *“If it [a brochure] was available, I'd put it in the waiting room. Because people always leaf through what's in the waiting room.” *	Convergent
			[PCP12, FG2, G]: *“Overall, I really like it online. I was previously in another practice and we had a cupboard full of brochures, but you have to manage them, then something is old, then you have to replace something and so on. And if you have it online, you can also print something for patients if they really only want something on paper. But then you always have access and, hmm, and everyone has access from the practice.”*	Expanding
	App for smartphone	34 (27.0)	[PCP2, I2, F]: *“But obviously, well, for older people, it [a smartphone app] doesn't work. And they don't have smartphones.”*	Convergent
			[PCP12, FG2, G]: *“I think a lot of older patients, my [my patients] write, most of them write emails, they google, they also deal with that [online media]. I could also imagine that they would also use apps.”*	Divergent
	Website	26 (20.6)	[PCP14, FG3, G]: *“Hmm, giving patients a link. I feel that the path is even longer. If you give them a brochure, they might find it in their handbag two weeks later. A link is probably less likely to be clicked on.”*	Convergent
	Documents for relatives/caregivers	63 (50.0)	[PCP14, FG3, G]: *“Hmm, yes, I think that [recommendations for sleep hygiene] would sometimes be good for the relatives too. Hmm, that you could just explain it or hand it [a brochure] over. So mostly we have the patients. But occasionally the daughter says, ‘Yes, my mother cannot anymore get enough sleep.’”*	Expanding
CONTENT	Explanations about risks and benefits of BSHs	112 (88.9)	[PCP5, I5, F]: *“Then indeed, ‘What are the side effects of, of benzos and Z-drugs?’. I think that's really important. Cognitive disorders, the risk of falling, of injuring oneself, I think that's really something that can affect patients.”*	Convergent
	Explanations about the discontinuation process	95 (75.4)	[PCP5, FG5, F] *“And then effectively tell them [the patients] that we're going to try to wean them off, and maybe give them some contact details of people who can help them with cognitive behavioral therapy.”*	Convergent
	Tapering schemes	92 (73.0)	[PCP3, I3, F]: *“If some schemes are deemed to work better than others, why not. I'm sure it could be a useful tool. And then, of course, you have to adapt it individually to each person.”*	Convergent
	Recommendations for sleep hygiene	101 (80.2)	[PCP5, I5, F] *“So I think we have to talk about hygiene, we have to talk about screens, we have to talk about light, we have to talk about meals, hmm, all that sort of things. (…) It's more in relation to behavioral aspects that we could have a benefit.”*	Convergent
	Testimonials	46 (36.5)	[PCP13, FG2, G]: *“So I think that [testimonials] is certainly interesting. But I don't know whether it's even more effective if, hmm, the case description is from someone you know. So there's often an emotional connection involved somehow. And if they [the patients] know, “I know this person, they can do it, then I can do it too.” I don't know how effective it would be if it was anonymous.”*	Expanding

*Abbreviations: BSHs* Benzodiazepines and other sedative hypnotic drugs, *CBT-I* Cognitive behavioral therapy for insomnia, *F* French, *FG* Focus group, *G* German, *I* Interview, *PCP* Primary care provider

**Table 3:** Meta-interference: Needs of PCPs (*N* = 126)

	**Variable**	**N (%)**	**Reflective quote**	**Meta-interference**
FORMAT	Online training	79 (62.7)	[PCP11, FG2, G]; *“Well, I find that [accreditable online training] very interesting.”*	Expanding
	In-person training	47 (37.3)	[PCP14, FG3, G]: *“And it [BSH deprescribing] probably, hmm, just, hmm, needs to be discussed why it's important. And then, how to approach that probably needs to be more workshop-like (…). Obviously, many of us don't know why it's worth doing it at all. And just how little use the drugs really are in the end for how many side effects they cause. I think that would shake us up. That would be a good thing*.”	Divergent
	Information on a website	65 (51.6)	[PCP13, FG2, G]: *“Hmm, I could, I would find some instruction or an informative site very helpful, yes.”*	Convergent
CONTENT	Practical recommendations for pharmacological and non-pharmacological treatment of sleep problems in older people with current BSH consumption	111 (88.1)	[PCP14, FG3, G]: *“And yes, I think I'm not good enough at explaining to people why it's counterproductive for them to take these substances. I think that's the beginning and if I don't manage that, then even if I have a good discontinuation plan, it's no use to me.”*	Expanding
			[PCP6, I6, F]: *“I think that, I think that the main recommendation is to say that you have to make a sleep diary and then do an exact investigation of what's going on.”*	Expanding
	Deprescription scheme for BSHs	86 (68.3)	[PCP 11, FG2, G]: *“So I think I would find it very helpful to get instructions on exactly how to proceed. Because I don't have much experience in this area.”*	Convergent
			[PCP 3, I3, F]: *“After that, what we primary care physicians really need, it’s like a recipe.”*	Convergent
	Principles of CBT-I for the treatment of sleep problems	77 (61.1)	[PCP 20, S, F]: *“What I found most useful was the CBT training day on sleep.”*	Convergent
	Implementation of CBT-I for the treatment of sleep problems in the primary care practice	72 (57.1)	[PCP6, I6, F]: *“I use, I use sleep restriction quite willingly. I use it myself as an internist.”*	Convergent
	PCP would like to complete CBT-I training if possible	74 (58.7)	[PCP13, FG2, G]: *“Yes, I would also be motivated [to complete CBT-I training]. Of course, it also depends on how time-consuming the training would be. But I think it's an important topic. I have, so, I feel that currently we are taking over a lot of psychotherapist work, hmm, and we have zero training.”*	Convergent
	Motivational interviewing for BSH discontinuation (e.g., videos/text providing examples of conversations about BSH discontinuation with patients)	33 (26.2)	[PCP 14, FG3, G]: *“Yes, or perhaps communicatively. To be honest, I rarely manage to get someone to stop smoking. So I think that's the challenge.”*	Divergent
			[PCP4, I4, F]: *“So it seems to me that motivational interviewing is something that is now widely taught and one masters more or less. It's always good to repeat it, but for me it's more about practical tools. For how to do it, when the person is motivated to listen. But how to get them to change their behavior. I think that's what would interest me.”*	Expanding
	Shared-decision-making tools for BSH discontinuation	52 (41.3)	[PCP10, FG1, G]: *“… and the second type [of tools] would also be like participatory decision-making when a person is in need and has the feeling that “Now I have to sleep again or it will be bad at work” or wherever. (…) And then, if you get into a position of refusal, then, hmm, I don't think you're really helping people. And I think it would be good to look for solutions with the people themselves, but I haven't seen any documentation on this yet. Where you could really discuss a participatory decision-making process with the advantages and disadvantages of the different options with them.”*	Divergent

*Abbreviations: BSHs* Benzodiazepines and other sedative hypnotic drugs, *CBT-I* Cognitive behavioral therapy for insomnia, *F* French, *FG* Focus group, *G* German, *I* Interview, *PCP* Primary care provider, *S* Survey

#### Preferences

PCPs were asked whether they preferred standardized materials, i.e., where the information and tapering schemes are similar for all patients, or customizable materials, where the information and tapering schemes can be individualized to each patient. Eighty-eight (69.8%) PCPs preferred customizable and 36 (28.6%) standardized materials for patients, while 2 (1.6%) PCPs had no preference.

#### Format

Eighty-seven (69.1%) PCPs mentioned they would find brochures for patients useful and that giving a brochure to the patients to read at home could facilitate deprescribing [PCP2, I2, F]: *“I mean, if there was something we could give our patients so that, in fact, we could already talk about it at the consultation. So that they can read their brochure or not. But it also tells us if the patient is a bit motivated. And then, afterwards, we can discuss it. It would probably save us time.”* Sixty-three (50.0%) PCPs wished documents for relatives, 62 (49.2%) materials for patients with cognitive impairment, and 62 (49.2%) flyers. Figures, apps or websites were thought as less useful.

#### Content

A clear preference was found for explanations about risks and benefits of BSHs, wished by 112 (88.9%) PCPs [PCP2, I2, F]: *“I think what would be important is the, the, that, it's the undesirable effects. So that we, so that they [the patients] understand why we have to change, obviously.”* A majority of PCPs also considered recommendations for sleep hygiene, explanations about alternative treatments and about the discontinuation process, as well as tapering schemes, as potentially helpful. Additionally, PCPs wished explanations about sleep physiology for their patients [PCP5, I5, F]: *“So, I think one of the main elements is to talk about sleep cycles and explain that these micro-awakenings are natural and difficult to avoid. I think there really needs to be this aspect of ‘What is normal sleep?’, and then, ‘What can we expect from sleep?’, and then, ‘What can't we expect too much of, let's say, with age?’”.* A table comparing the effectiveness of the different treatments or testimonials were wished by few PCPs, 50 (39.7%) and 46 (36.5%), respectively.

### PCP needs

#### Format

Concerning useful resources for PCPs, 79 (62.7%) preferred online training, followed by online documents, information on a website and exchange with colleagues. In-person training, printed documents and apps for smartphone were wished by less PCPs. Regarding training in general, PCPs mentioned that sleep problems were just one relevant topic among lots of others [PCP12, FG2, G]: *“I don't know if we all really need to do so much training now on how to reduce it [BSH use] exactly. But, hmm, maybe a few basics. (…) But we have to, it's so varied, we have to be fit in so many topics and so I wouldn't want to spend half a day just talking about sleep problems, hmm, or.”*

#### Content

Practical recommendations for pharmacological and non-pharmacological treatment of sleep problems in older people with current BSH consumption were wished by 111 (88.1%) PCPs and deprescribing schemes by 86 (68.3%) [PCP 11, FG2, G]: *“So, I think I would find it very helpful to get instructions on exactly how to proceed. Because I don't have much experience in this area.”* Concerning CBT-I, 72 (57.1%) PCPs wished its implementation into primary care practice. Seventy-seven (61.1%) said they would be willing to receive information about CBT-I and 74 (58.7%) to complete CBT-I training if it was offered. A minority of PCPs (46, 36.5%) wished recommendations for follow-up and information about motivational interviewing (33, 26.2%). PCPs said, a list of therapists offering CBT-I for sleep problems for older adults using BSHs would be helpful [PCP5, I5, F]: *“Well, maybe a list of psychologists who, who willingly take on this type of patients.”*

## Discussion

In this mixed-methods study, we assessed barriers and facilitators as well as needs of PCPs to BSH deprescribing. The identified TDF constructs mostly confirmed existing literature [12, 13]. Main barriers to deprescribing included patient and PCP lack of knowledge on BSH effects and side effects, PCP lack of education on BSH deprescribing and treatment of sleep problems, patient lack of motivation, PCP lack of time, limited access to CBT-I, and absence of public dialogue on BSHs. Facilitators included informing patients about BSH side effects and deprescribing start during hospitalization. The main PCP needs were practical recommendations for pharmacological and non-pharmacological treatment of sleep problems and deprescribing schemes. For their patients, they wished brochures containing explanations about risks and benefits of BSHs, sleep hygiene and sleep physiology, alternative treatments, discontinuation process and tapering schemes.

Initiating deprescribing of inappropriate BSHs during hospitalization was mentioned as a potential facilitator. This confirms existing literature, showing hospitalization to be an opportunity to review medication and initiate deprescribing [20, 21]. However, ensuring continuity could be difficult and PCPs mentioned their patients often resumed BSH use after discharge, using the medication they still had at home, the prescription they had before hospitalization, or asking PCPs for a new prescription. Previous research identified concerns about lack of post-discharge follow-up and disagreement between hospitalists and PCPs [18, 20]. This could be improved by enhancing communication around discharge between hospital internists and PCPs, for example by phone contact or electronic communication, and implementing structured care coordination [22]. It is particularly important that hospital internists communicate to PCPs the shared decision made with the patients regarding deprescribing during hospitalization. Further, PCPs suggested promoting public dialogue on BSHs to support deprescribing. However, public campaigns seem only moderately effective to reduce BSH consumption [23].

Not all PCPs knew where to refer patients for the treatment of sleep problems and CBT-I. Further, lack of CBT-I therapists and CBT-I costs were mentioned as barriers. Limited access to psychotherapy and often insufficient insurance coverage is a widespread problem globally [24, 25]. Absence of psychotherapy reimbursement was previously described as a barrier to deprescribe BSHs [26]. Providing CBT-I therapists contact details to PCPs, PCP training in CBT-I and self-help CBT-I could help address this issue [27, 28].

Regarding PCP opinions on patient materials, they preferred brochures over online material, to avoid limiting access. Research has shown that patient information leaflets could decrease the number of repeat visits to PCPs and therefore save time but should in no way substitute oral information [29]. Also, the timing of information is relevant [29]. Providing information prior to the consultation could be beneficial and give patients time to consider the benefits [12, 30]. Using patient brochures to support BSH deprescribing was shown to be effective in the EMPOWER trial which tested a pharmacist-led intervention [31]. Generalizability of this trial is however limited because the implementation in pharmacies might not work in healthcare systems, where pharmacists do not conduct medication review or follow patients on a regular basis, especially if physician drug dispensing is allowed, like in Switzerland. Therefore, multilevel interventions including patient information brochures accounting for country-specific differences should be developed.

PCPs requested online training covering practical recommendations for pharmacological and non-pharmacological treatment of sleep problems in older people currently using BSH. This is consistent with the reported lack of education on sleep problems and BSH deprescribing during medical school, residency and continuing education. Previous research showed that the term “deprescribing” was unfamiliar to medical students and that physician education on BSH deprescribing and insomnia treatment should be reinforced [26, 32, 33]. Online modality of continuous medical education was found to provide flexibility of access in terms of time and geographic location [34]. Furthermore, PCPs wished to complete CBT-I training to implement it in their practice. These findings underline the importance of integrating training on CBT-I and treatment of sleep problems at medical school and in post-graduate training.

### Strengths and limitations

This study has several strengths. First, the mixed methods design with deductive and inductive approach allowed a more comprehensive understanding. Second, the inclusion of PCPs of different practice types (single/group practice) and regions (rural/city, French-/German-speaking) increases result generalizability.

We must acknowledge some limitations. First, as PCPs who participated to the study are potentially more intrigued to deprescribe BSHs, their opinions might not reflect those of all PCPs. Second, the study was conducted in a single country. Nevertheless, assessing local factors is required for successful implementation. Third, we did not power our study to conduct additional analyses according to baseline characteristics such as age, work status or years of experience. Therefore, except for language, other elements of variation of sample were not considered. Finally, the conduction of both FGs and interviews can be both a limitation and a strength. On the one hand, while FGs might have allowed new thoughts to emerge by discussing with colleagues, participants might also have been hesitant to express divergent opinions in front of colleagues. On the other hand, interviews allowed a more intimate setting to express opinions but the lack of exchanges and stimulation by colleagues might have limited PCP reflections.

## Conclusion

The identification of barriers and facilitators to BSH deprescribing and particularly of PCP needs to support BSH deprescribing can help develop appropriate materials to reduce the use of BSHs and their adverse effects, as well as training for medical students and board-certified physicians.

### Supplementary Information

Supplementary Material 1: Appendix Table 1. Survey results – Patient material. Appendix Table 2. Survey results Needs of PCPs. Appendix Table 3. TDF domains and constructs used for coding

Supplementary Material 2. Survey for PCPs

Supplementary Material 3. Interview guide for the Focus groups and interviews with PCPs

## Data Availability

Data are available by the corresponding author upon reasonable request.

## Abbreviations

BSH: Benzodiazepine and other sedative hypnotic drug
CBT-I: Cognitive behavioral therapy for insomnia
F: French
FG: Focus group
G: German
HCP: Healthcare professional (specialized physician and/or non-physician therapist)
I: Interview
PCP: Primary care provider
S: Survey
TDF: Theoretical Domains Framework
y: Years
