# Supplementary material for: Barriers, facilitators and needs to deprescribe benzodiazepines and other sedatives in older adults: a mixed methods study of primary care provider perspectives
Source: BMC Geriatr. 2024 May 4;24:396. doi: 10.1186/s12877-024-05027-9 (PMC11069272; doi:10.1186/s12877-024-05027-9)
Supplement: Supplementary file 1 — Supplementary Material 1: Appendix Table 1. Survey results – Patient material. Appendix Table 2. Survey results Needs of PCPs. Appendix Table 3. TDF domains and constructs used for coding [file 12877_2024_5027_MOESM1_ESM.pdf]

Additional File 1: Tables

Appendix Table 1. Survey results (N=126) – Patient material

VARIABLE	N (%)
**Preference**	
Customizable material	88 (69.8)
Standardized material	36 (28.6)
No preference	2 (1.6)
**Format**	
Flyers	62 (49.2)
Brochures	87 (69.1)
App for smartphone	34 (27.0)
Videos	20 (15.9)
Website	26 (20.6)
Schemes	54 (42.9)
Figures	22 (17.5)
Documents for relatives/caregivers	63 (50.0)
Material adapted for people with cognitive impairment	62 (49.2)
Material adapted for people with visual impairment	46 (36.5)
**Content**	
Explanations about risks and benefits of BSHs	112 (88.9)
Explanations about the discontinuation process	95 (75.4)
Tapering schemes	92 (73.0)
Explanations about treatment of withdrawal symptoms	67 (53.2)
Explanations about alternative treatments of sleep problems	100 (79.4)
Table/figure comparing the effectiveness of the different treatments for sleep problems	50 (39.7)
Recommendations for sleep hygiene	101 (80.2)
Testimonials	46 (36.5)

Abbreviations: BSHs = Benzodiazepines and Sedative Hypnotics.

Appendix Table 2. Survey results (N=126) – Needs of PCPs

VARIABLE	N (%)
**Format**	
Online training	79 (62.7)
In-person training	47 (37.3)
Online documents	77 (61.1)
Printed documents	43 (34.1)
Information on a website	65 (51.6)
Exchange with colleagues (e.g., in the context of quality circles)	57 (45.2)
App for smartphone	28 (22.2)
**Content**	
Practical recommendations for (pharmacologic and non-pharmacologic methods) treatment of sleep problems in older people with current BSH consumption	111 (88.1)
Practical recommendations for (pharmacologic and non-pharmacologic methods) treatment of sleep problems in older people without current BSH consumption	72 (57.1)
Recommendations for follow-up of patients during the discontinuation process of BSHs	46 (36.5)
Indications and contraindications for the prescription and deprescription of BSHs	62 (49.2)
Deprescription Scheme for BSHs	86 (68.3)
Principles of CBT-I for the treatment of sleep problems	77 (61.1)
Implementation of CBT-I for the treatment of sleep problems in the primary care practice	72 (57.1)
PCP would like to complete CBT-I training if possible	74 (58.7)
Practical recommendations for the treatment of BSH withdrawal symptoms	70 (55.6)
Motivational interviewing for BSH discontinuation (e.g., videos/text providing examples of conversations about BSH discontinuation with patients)	33 (26.2)
Shared-decision-making tools for BSH discontinuation	52 (41.3)

Abbreviations: BSH = Benzodiazepine and Sedative Hypnotic; CBT-I = cognitive behavioral therapy for insomnia; PCPs = primary care providers.

Appendix Table 3. TDF domains and constructs used for coding

Domains	Constructs
Knowledge	Knowledge about condition/scientific rationale
	Schemas + mindsets + illness representations
	Procedural knowledge
Skills	Competence/ability/skill assessment
	Practice/skills development
Beliefs about capabilities	Perceived competence
	Self-confidence/professional confidence
Beliefs about consequences	Outcome expectancies
	Consequents
	Unrealistic optimism
Motivation and goals	Goal target/setting
	Goal priority
	Intrinsic motivation
	Transtheoretical model and stages of change
Environmental context and resources	Resources/material resources (availability and management)
Social influences	Social support
	Social/group norms
	Social pressure
	Inter-group conflict
	Social comparisons
Emotion	Affect
	Anticipated regret
	Fear
	Anxiety/depression
Behavioral regulation	Goal/target setting
	Self-monitoring
	Moderators of intention-behavior gap
	Barriers and facilitators
Nature of behaviors	Routine/automatic/habit
	Breaking habit
	Direct experience/past behavior
	Representation of tasks

Abbreviations: TDF = Theoretical Domains Framework
