# Supplementary material for: Barriers, facilitators and needs to deprescribe benzodiazepines and other sedatives in older adults: a mixed methods study of primary care provider perspectives
Source: BMC Geriatr. 2024 May 4;24:396. doi: 10.1186/s12877-024-05027-9 (PMC11069272; doi:10.1186/s12877-024-05027-9)
Supplement: Supplementary file 2 — Supplementary Material 2. Survey for PCPs [file 12877_2024_5027_MOESM2_ESM.pdf]

Additional File 2. Survey for PCPs

Introduction

The present survey aims to identify barriers and enablers to the discontinuation of benzodiazepines and sedative/hypnotic drugs in older people (65 years or older) with sleep problems from the perspective of primary care physicians. In addition, the survey aims to identify the support needs of GPs in assisting patients to discontinue benzodiazepines.

Here are important definitions or terms that are used throughout the survey. Please carefully read them before starting to answer the questions.

BSHs – Benzodiazepine and Sedative Hypnotics, namely benzodiazepines and z-drugs such as:

Alprazolam (Xanax®), Bromazepam (Lexotanil®), Clobazam (Urbanyl®, Diacomit ®), Clonazepam (Rivotril®, Tranxilium®), Diazepam (Psychopax®, Valium®), Flurazepam (Dalmadorm®), Lorazepam (Temesta®), Lormetazepam (Loramet®), Oxazepam (Anxiolit®, Seresta®), Nitrazepam (Mogadon®), Prazepam (Demetrin®), Triazolam (Halcion®), Zolpidem (Stilnox®, Zoldorm®), Zopiclone (Imovane®).

***BSH Discontinuation (discontinuing BSHs)* –** it is the process of tapering (i.e., gradual dose reduction) and finally stopping the BSH. This includes several subactions such as checking eligibility to deprescribe, engaging with the patient (providing information, shared decision making), implementing progressive tapering, selecting alternatives whenever needed, and following up.

***To engage patients* -** to cause someone to become interested or involved in an activity, or to attract someone's interest; in the context of this survey, it means to start the discussion with patients about the discontinuation of BSH and motivate them to discontinue.

This survey is divided into 4 sections:

1) 9 questions on demographic parameters, professional experience and daily work routine.
2) 3 questions on the need for material for patients.
3) 3 questions about your needs as a family doctor.
4) 3 open questions for you to add additional information.

Section 1: Demographic parameters, professional experience and daily work routine

1. Which age category do you belong to?

- ☐ 30 years old or younger
- ☐ 31 – 40 years old
- ☐ 41 – 50 years old
- ☐ 51 – 60 years old
- ☐ 61 years old or older

2. What is your gender?
 Male
 Female
 Other
 Prefer not to answer

3. What kind of practice do you work in?
 Alone (in the office on my own)
 Monodisciplinary practice, i.e., a practice with primary care physicians only
 Multidisciplinary practice with primary care physicians and non-physician therapists
 Multidisciplinary practice with primary care physicians and other specialists +/- non-physician therapists
 Other, please specify: ___________________

4. Are you independent or employed?
 Independent
 Employed

5. How many years of clinical experience (in general) do you have?
 Less than 5 years
 5 – 9 years
 10 – 14 years
 15 – 19 years
 20 years or more

6. Have you ever discontinued a BSH in older adults with sleep problems?
 YES
 NO

7. Do you routinely consider BSH discontinuation in older adults taking a BSH for sleep problems?
 YES
 NO

8. Do you know where to refer your patients for treatment of sleep problems if needed?
 YES: please specify: ___________________
 NO

9. Do you know where to refer your patients for treatment of sleep problems using cognitive behavioral therapy for insomnia, if needed?
 YES: please specify: ___________________
 NO

Section 2: Material for patients to support BSH discontinuation

In this section, we are interested in materials that you think would be useful for patients to support discontinuation of BSHs.

10. What kind of patient material do you consider useful / appropriate (multiple answers possible)?
    - ☐ Flyers
    - ☐ Brochures
    - ☐ App for smartphone
    - ☐ Videos
    - ☐ Website
    - ☐ Schemes
    - ☐ Figures
    - ☐ Documents for relatives / caregivers
    - ☐ Material adapted for people with cognitive impairment
    - ☐ Material adapted for people with visual impairment
    - ☐ Other: please specify: __________________

11. What content for patient material do you consider useful (multiple answers possible)?
    - ☐ Explanations about risks and benefits of BSHs
    - ☐ Explanations about the discontinuation process
    - ☐ Tapering schemes
    - ☐ Explanations about treatment of withdrawal symptoms
    - ☐ Explanations about alternative methods for treating sleep problems
    - ☐ Table/figure comparing the effectiveness of different methods for treating sleep problems
    - ☐ Recommendations for sleep hygiene
    - ☐ Testimonials
    - ☐ Other: please specify: __________________

12. What type of patient material would you prefer?
    - ☐ Customizable material (e.g., for tapering schemes)
    - ☐ Standardized material (not customizable)

<u>Section 3: Own support needs as a primary care physician to support patients in discontinuing BSHs</u>

In this section, we are interested in what would be helpful to you as a primary care physician to support the discontinuation of BSHs.

13. What forms of resources would be helpful to you personally (multiple answers possible)?
    - ☐ Online training
    - ☐ In-person training
    - ☐ Online documents
    - ☐ Printed documents
    - ☐ Information on a website
    - ☐ Exchange with colleagues (e.g., in the context of quality circles)
    - ☐ App for smartphone
    - ☐ Other: please specify: __________________

14. Which content would be helpful for you personally (several answers possible)?
    - ☐ Practical recommendations for (pharmacologic and non-pharmacologic) treatment of sleep problems in older people with current BSH consumption.
    - ☐ Practical recommendations for (pharmacological and non-pharmacological) treatment of sleep problems in older people WITHOUT current BSH consumption.
    - ☐ Recommendations for follow-up of patients during the discontinuation process of BSHs.
    - ☐ Indications and contraindications for the prescription and discontinuation of BSHs.
    - ☐ Principles of cognitive behavioral therapy for sleep problems.
    - ☐ Implementing cognitive behavioral therapy for sleep problems in primary care practice.
    - ☐ Practical recommendations for treating BSH withdrawal symptoms.
    - ☐ Motivational interviewing for BSH discontinuation (e.g., videos/text providing examples of conversations about BSH discontinuation with patients).
    - ☐ Shared decision-making tools for BSH discontinuation.
    - ☐ Other: please indicate: ___________________

15. If the opportunity was available: would you like to complete training in cognitive behavioral therapy for sleep problems?
    - ☐ YES
    - ☐ NO

<u>Section 4: Additional comments</u>

16. Are there any other barriers to BSH discontinuation in older adults with sleep problems that you would like to add?

17. Are there any other enablers to BSH discontinuation in older adults with sleep problems that you would like to add?

18. Is there anything else you would like to share about BSH discontinuation in older adults with sleep problems?

