# Supplementary material for: Barriers, facilitators and needs to deprescribe benzodiazepines and other sedatives in older adults: a mixed methods study of primary care provider perspectives
Source: BMC Geriatr. 2024 May 4;24:396. doi: 10.1186/s12877-024-05027-9 (PMC11069272; doi:10.1186/s12877-024-05027-9)
Supplement: Supplementary file 3 — Supplementary Material 3. Interview guide for the Focus groups and interviews with PCPs [file 12877_2024_5027_MOESM3_ESM.pdf]

Additional File 3. Interview guide for the Focus groups and interviews with PCPs

Introduction

Hello,

- Thank you for agreeing to participate to this interview. [Interviewer presents themselves]. The aim of this interview is to hear your views, perspectives and opinions on the deprescription of sleeping pills in elderly people who take them for sleep problems, and also to discuss what might help you and your patients in this process. By sleeping pills, we refer only to benzodiazepines and Z-drugs, such as Valium®, Temesta®, Seresta® or Zolpidem.
- This interview is part of the preparatory phase of an international project funded by Horizon Europe and the College of Primary Care Medicine about the deprescription of sleeping pills in the elderly. This is the BE-SAFE study.
- There are no right or wrong answers, the approach is qualitative and exploratory.
- We record the discussion so that we can then transcribe and evaluate it. Your name will not appear anywhere, and we will not spread the audio content.
- Do you have a question before moving on?

Questions

1) Could you tell us about a situation where you wanted to deprescribe a sleeping pill and it was difficult or impossible? What were the obstacles and difficulties?
   If necessary, guide with examples:
   a) *Fear of side effects / withdrawal on stopping*
   b) *Lack of alternatives*
   c) *Patient refusal / patient satisfied with treatment / dependence*
   d) *Lack of time*
   e) *No reason to stop (no side effects, no interactions)?*
2) Can you tell us about a situation where the deprescription of a sleeping pill went well? What facilitated the deprescription (patient factors, your own factors, other factors)?
   If necessary, guide with examples:
   a) *Patient's wish*
   b) *Alternative treatment (CBT-I (cognitive behavioral therapy for insomnia), melatonin)*
   c) *Parallel treatment by sleep specialists*
   d) *Sleep hygiene recommendations*
   e) *Recommendations / tools for deprescribing*
   f) *Patient testimonies of success*
   g) *Algorithms / visual representation / decision support tools for patients*
   h) *Motivational interviewing techniques*
3) What do you think of the training you have (or haven't) received, are receiving or have available about managing sleep disorders in the elderly and the use and deprescription of sleeping pills?

4) What resources would be useful to you as a physician, and in what form (online/paper/...)?

    If necessary, give examples:

    a) Motivational interviewing training
    b) CBT-I training
    c) Access to specialists
    d) Recommendations with deprescription schemes
    e) Decision-support tools

5) What resources would you find useful for your patients, and in what form (online/paper/...)?

    If necessary, give examples:

    a) Visual schemes
    b) Decision-support tools
    c) CBT-I resources
    d) Sleep hygiene materials
    e) Testimonials

6) 6) Are there any other points that are important to you and that you'd like to mention about the deprescription of sleeping pills?

We've come to the end of this interview. Thank you once again for your participation. I'll send you the billing document to fill in.
